# Effect of Functional Endoscopic Sinus Surgery on Quality of Life in Patients With Chronic Rhinosinusitis: A Nonrandomized Trial

**DOI:** 10.7759/cureus.114538

**Published:** 2026-08-14

**Authors:** Nitin V Deosthale, Vallabh V Kulkarni, Sonali P Khadakkar, Jini Telrandhe, Fahad Idrees Shaikh, Nishka Tiwari

**Affiliations:** 1 Otolaryngology - Head and Neck Surgery, N.K.P. Salve Institute of Medical Sciences and Research Centre and Lata Mangeshkar Hospital, Nagpur, IND; 2 Medicine, N.K.P. Salve Institute of Medical Sciences and Research Centre and Lata Mangeshkar Hospital, Nagpur, IND; 3 Medical Education, N.K.P. Salve Institute of Medical Sciences and Research Centre and Lata Mangeshkar Hospital, Nagpur, IND

**Keywords:** chronic rhinosinusitis, crsnnp, crswnp, fess, functional endoscopic sinus surgery, lund-kennedy score, nasal polyps, quality of life, snot-22

## Abstract

Background: Chronic rhinosinusitis (CRS) is a persistent inflammatory disorder of the nose and paranasal sinuses characterized by substantial symptom burden and disease-specific impairment of quality of life. Functional endoscopic sinus surgery (FESS) is offered to patients who remain symptomatic despite adequate medical therapy, and outcome assessment should encompass both patient-reported and objective endoscopic measures.

Objective: The aim of this study was to evaluate the effect of FESS on disease-specific quality of life and objective endoscopic status in patients with CRS and to compare preoperative disease burden between CRS phenotypes.

Methods: This nonrandomized trial enrolled 96 patients diagnosed with CRS who underwent FESS at a tertiary care center. Baseline demographic data, CRS phenotype (with or without nasal polyps), presenting symptoms, comorbidities, and anterior rhinoscopy findings were recorded. Quality of life was assessed using the validated 22-item Sino-Nasal Outcome Test (SNOT-22) preoperatively and at three and six months postoperatively. Objective endoscopic status was evaluated using the Lund-Kennedy endoscopic score at identical time points. Preoperative scores were compared between CRS with nasal polyps (CRSwNP) and CRS without nasal polyps (CRSsNP) using Welch's independent-samples t-test. Longitudinal changes in scores were assessed using the Friedman repeated-measures test, and categorical severity distributions were compared with the chi-square test.

Results: The mean age was 39.96 ± 10.39 years, and 63 (65.6%) patients were male. Nasal blockage was present in 93 (96.9%) patients, nasal discharge in 85 (88.5%), and smell disturbance in 67 (69.8%). CRSwNP was identified in 59 patients (61.5%). Patients with CRSwNP had a significantly higher mean preoperative SNOT-22 score than those with CRSsNP (45.20 ± 12.87 vs. 39.22 ± 11.71; p = 0.021) and a significantly higher mean preoperative Lund-Kennedy score (9.92 ± 2.44 vs. 7.41 ± 2.49; p < 0.001). Following FESS, the overall mean SNOT-22 score decreased from 42.90 ± 12.72 preoperatively to 12.50 ± 7.98 at three months and 9.65 ± 6.33 at six months (p < 0.001). The proportion of patients in the severe SNOT-22 category (score >50) decreased from 30 (31.2%) preoperatively to 0 (0%) at both postoperative assessments, while the proportion in the mild category increased from 4 (4.2%) to 90 (93.8%) by six months. The mean Lund-Kennedy score decreased from 8.95 ± 2.74 at baseline to 3.61 ± 2.23 at three months (59.6% reduction) and 1.16 ± 1.42 at six months (87.1% reduction; p < 0.001).

Conclusion: FESS significantly and consistently improved both disease-specific quality of life and objective endoscopic findings in patients with medically refractory CRS. Patients with nasal polyps had a higher disease burden before surgery, yet both polyp and non-polyp groups experienced meaningful postoperative improvements. Using both the SNOT-22 questionnaire and the Lund-Kennedy endoscopic score offers a thorough and clinically relevant method to assess surgical outcomes in CRS.

## Introduction

Chronic rhinosinusitis (CRS) is a chronic inflammatory condition of the nose and paranasal sinuses. It is characterized by persistent sinonasal symptoms lasting for more than 12 weeks, accompanied by objectively confirmed mucosal disease. It is a major concern in otorhinolaryngology due to its high prevalence, recurrent nature, resource-intensive management, and significant effect on day-to-day functioning. Although rarely life-threatening, CRS substantially reduces quality of life by causing persistent symptoms, frequent medical visits, extended medication use, and adverse effects on sleep, smell, work performance, and emotional health [1,2]. CRS represents a considerable global health burden on affected populations worldwide. Epidemiological studies have demonstrated regional variation in its prevalence, with estimates of approximately 10.9% in Europe, 4.5-12% in the United States, and around 6.8% in Asia [3-5].

Clinically, the condition is broadly classified into two phenotypes: CRS with nasal polyps (CRSwNP) and CRS without nasal polyps (CRSsNP). These two differ in their clinical manifestations, underlying inflammatory mechanisms, endoscopic appearance, association with comorbid conditions such as asthma and allergy, and response to treatment. Diagnosis largely depends on symptom assessment and objective findings from nasal endoscopy and/or radiological evaluation, as symptom severity alone is not sufficient to reflect the extent of disease [1,6,7].

The pathogenesis of CRS is quite intricate and multifactorial. Prolonged inflammation may arise from impaired mucociliary clearance, blocked sinus openings, swelling of the osteomeatal complex, recurrent microbial infections, allergic reactions, and anatomical abnormalities. Together, these factors interfere with the normal ventilation and drainage of the paranasal sinuses, causing mucus retention, mucosal thickening, polyp formation, and sustained inflammation. The chronic and recurrent nature of CRS significantly affects patients' quality of life, producing symptoms such as nasal obstruction, nasal discharge, facial pressure, headache, loss of smell, snoring, and prolonged sleep disturbances [6-8].

Management of CRS begins with medical treatment aimed at controlling mucosal inflammation, improving sinus drainage, treating underlying infections, and addressing contributing factors such as allergies and structural issues. Despite appropriate treatment, some patients remain symptomatic. In such cases, functional endoscopic sinus surgery (FESS) is considered the treatment of choice. FESS re-establishes normal sinus ventilation and mucociliary clearance by enlarging the natural sinus drainage openings while preserving healthy mucosa. The surgical extent is customized based on the disease location and may involve procedures such as uncinectomy, middle meatal antrostomy, anterior and posterior ethmoidectomy, frontal recess clearance, and sphenoidotomy, depending on the clinical indication [9-11].

Outcome assessment following FESS should extend beyond confirming operative patency or interpreting radiological appearance. Although endoscopic evaluation performed by the surgeon is important, the Lund-Kennedy endoscopic score provides a standardized and reproducible way to measure mucosal swelling, discharge, polyps, scarring, and crusting through repeated assessments [12]. Because CRS is fundamentally a patient-centered condition, the success of treatment should therefore be evaluated not only by objective findings but also by meaningful patient experiences, including significant improvements in breathing, sleep, sense of smell, and overall functional ability. Therefore, validated patient-reported outcome measures have become essential in both CRS research and routine clinical practice.

The Sino-Nasal Outcome Test (SNOT-22) is a 22-item disease-specific quality-of-life instrument and one of the most commonly used tools in CRS assessment. It encompasses rhinological, extra-nasal, ear and facial, sleep, and psychological domains; is straightforward to administer; and has demonstrated sensitivity to clinically meaningful change following treatment [13,14]. Prior studies have confirmed that FESS yields significant improvements in symptoms and quality of life among patients with CRS, although the magnitude of benefit varies with baseline severity, disease phenotype, comorbid conditions, and the duration of follow-up [15-18].

Data from Indian tertiary care centers are particularly relevant, as patients in this context may present after prolonged symptom duration, have received repeated empirical therapies before referral, and may differ from Western cohorts in environmental exposures, healthcare access, and inflammatory profiles. Therefore, the present study was designed to assess the effect of FESS on disease-specific quality of life using the SNOT-22 questionnaire and to evaluate objective endoscopic change using the Lund-Kennedy score in patients with CRS managed at a tertiary care center. Baseline quality of life and endoscopic burden were compared between CRSwNP and CRSsNP phenotypes, and changes in both outcome measures were tracked serially at 3 and 6 months postoperatively.

The effect of CRS on quality of life is broad and extends beyond nasal symptoms alone. Persistent nasal obstruction disrupts sleep patterns, encourages mouth breathing, and reduces exercise tolerance. Discharge and postnasal drip cause social embarrassment and frequent throat irritation. Olfactory dysfunction negatively affects appetite, safety, and emotional health. Although radiological scores accurately reflect the anatomical extent of the disease, they often fail to reflect its functional and symptomatic burden. Consequently, some patients with limited radiological findings may report marked sleep disturbances or facial pain, whereas others with extensive mucosal disease experience relatively milder symptoms. This discrepancy has led to a greater emphasis on incorporating validated patient-reported outcome measures alongside objective endoscopic findings when evaluating treatment success.

The timing of postoperative outcome assessment is also clinically important. Early evaluations may be influenced by temporary postoperative changes, such as crust formation, mucosal edema, and the need for debridement, which can affect the interpretation of results. In contrast, assessments performed at later follow-up reflect mucosal recovery and restoration of normal sinus ventilation more accurately. A follow-up period of approximately three months primarily demonstrates early clinical improvement, while a six-month review offers a clear and sustained recovery after postoperative healing. Consequently, serial evaluation at both intervals provides a more comprehensive understanding of recovery than a single follow-up visit.

The inherent variability in CRS phenotypes makes the interpretation of surgical outcomes challenging. Patients with the same diagnosis may differ significantly in inflammatory endotype, polyp load, allergic sensitization, asthma severity, prior medication use, and anatomical obstruction. These variations influence both initial symptom severity and postoperative outcomes. Therefore, studies that account for disease phenotype, associated comorbidities, and objective endoscopic findings generate more clinically meaningful insights. In the present study, the CRSwNP and CRS without nasal polyps (CRSsNP) subgroups were analyzed separately for baseline SNOT-22 and Lund-Kennedy scores to determine the additional burden of polypoidal disease.

In the Indian healthcare setting, postoperative quality-of-life assessment is particularly important for patient counseling. Many patients seek surgical referral only after experiencing long-standing symptoms and may have expectations ranging from complete cure to concerns about disease recurrence. Performing a structured pre- and postoperative SNOT-22 assessment helps the clinician to communicate anticipated symptomatic improvement, while serial endoscopic scoring allows surgeons to monitor healing, detect residual edema, or identify early recurrence. Using both tools together can improve follow-up communication, support evidence-based decisions regarding continuation of postoperative medical therapy, and aid in institutional audits and quality improvement.

## Materials and methods

Study design and setting

This nonrandomized, hospital-based prospective study was conducted in the Department of Otorhinolaryngology, NKP Salve Institute of Medical Sciences and Research Center and Lata Mangeshkar Hospital, Nagpur, Maharashtra, India, from May 1, 2024, to December 31, 2025. Patients diagnosed with chronic rhinosinusitis (CRS) who were scheduled to undergo functional endoscopic sinus surgery (FESS) after failure of adequate medical management were recruited consecutively. The diagnosis of CRS was established based on persistent sinonasal symptoms for more than 12 weeks together with objective findings on nasal endoscopy and/or computed tomography of the paranasal sinuses. The study was approved by the Institutional Ethics Committee of NKP Salve Institute of Medical Sciences & Research Center and Lata Mangeshkar Hospital, Nagpur (IEC Reference No. 19/2024; approval date: April 18, 2024). Written informed consent was obtained from all participants before enrollment.

Sample size calculation

The sample size was calculated using the variability in SNOT-22 scores reported by Kurien et al. The required sample size was estimated using the formula:

$n = \frac{Z^{2}(1&minus;&alpha;/2)\times \mathrm{SD}^{2}}{L^{2}}$

where Z represents the standard normal variate corresponding to the desired confidence level, SD is the standard deviation obtained from the reference study, and L is the relative precision fixed at 20% of the mean SNOT-22 score. Based on this calculation, the minimum required sample size was 96 participants. Accordingly, 96 eligible patients were enrolled in the present study.

Participants

A total of 96 patients undergoing FESS were enrolled. Inclusion criteria comprised patients of either sex with a diagnosis of CRS who were symptomatic despite appropriate medical management and who were considered suitable candidates for FESS. Patients were classified as CRSwNP or CRSsNP according to the presence or absence of nasal polyps on clinical and endoscopic assessment.

Patients were excluded if they had acute rhinosinusitis alone, sinonasal malignancy, invasive fungal rhinosinusitis, a major systemic contraindication to surgery, refusal of informed consent, or incomplete follow-up data.

Data collection

Demographic characteristics, symptom duration, presenting complaints, comorbidities, anterior rhinoscopy results, and CRS phenotype were systematically documented using a structured proforma. The preoperative assessment involved a thorough history of nasal obstruction, nasal discharge, olfactory issues, recurrent upper respiratory infections, headaches, sneezing, snoring, nosebleeds, and related ear or throat symptoms. Comorbid conditions such as asthma, diabetes mellitus, hypertension, and thyroid dysfunction were also recorded, given their potential impact on symptom severity, perioperative planning, and postoperative management.

Surgical procedure

All patients underwent FESS following standard endoscopic guidelines. The surgery's extent was tailored according to disease distribution. All patients underwent FESS under general anesthesia using 0° and 30° rigid nasal endoscopes. The extent of surgery was individualized according to the distribution and severity of disease. Surgical procedures included uncinectomy, middle meatal antrostomy, anterior and/or posterior ethmoidectomy, frontal recess clearance, and sphenoidotomy, wherever indicated. The primary objectives of surgery were to remove diseased tissue, restore normal sinus ventilation and drainage, and preserve healthy mucosa whenever feasible. Standard postoperative management included saline nasal irrigation, topical intranasal corticosteroids, antibiotics when clinically indicated, and scheduled endoscopic follow-up according to institutional protocol.

Outcome measures

The primary patient-reported outcome was quality of life, measured using the SNOT-22 questionnaire administered before surgery and at three and six months postoperatively. Each of the 22 items is scored from 0 to 5, producing a total score ranging from 0 to 110, where lower scores indicate better quality of life. For descriptive analysis, SNOT-22 severity was categorized as mild (score ≤20), moderate (score 21-50), and severe (score >50). The original English version of the SNOT-22 questionnaire was used without modification as the patient-reported outcome measure.

The objective endoscopic outcome was the Lund-Kennedy endoscopic score, assessed preoperatively and at three and six months postoperatively. The Lund-Kennedy endoscopic score was calculated by evaluating each nasal cavity separately for five parameters: polyps, edema, discharge, scarring, and crusting. Each parameter was scored from 0 to 2, and the bilateral scores were summed to obtain a total score ranging from 0 to 20. Higher scores indicate more severe endoscopic disease, whereas lower scores reflect improved mucosal healing and better postoperative endoscopic status. This instrument is feasible to perform repeatedly in an outpatient setting and reflects both the extent of residual disease and the degree of postoperative mucosal recovery.

Statistical analysis

Data were entered into Microsoft Excel (Microsoft Corporation, Redmond, Washington) and analyzed using IBM SPSS Statistics for Windows, Version 20 (Released 2011; IBM Corp., Armonk, New York). Continuous variables were expressed as mean ± standard deviation (SD), while categorical variables were presented as frequencies and percentages. Baseline SNOT-22 and Lund-Kennedy scores between the CRSwNP and CRSsNP groups were compared using Welch’s independent-samples t-test. Changes in SNOT-22 and Lund-Kennedy scores over time were analyzed using the Friedman repeated-measures test. Differences in categorical SNOT-22 severity distributions were analyzed using the chi-square test. A p-value <0.05 was considered statistically significant.

## Results

Baseline demographic and clinical profile

A total of 96 patients with CRS who underwent FESS were included in the analysis. The mean age was 39.96 ± 10.39 years, with a median of 38.5 years and a range of 19-67 years. The largest age group was 31-40 years (34.4%), followed by 41-50 years (26.0%). Male patients constituted 63 (65.6%) of the cohort and female patients 33 (34.4%), yielding a male-to-female ratio of approximately 1.9:1. The mean duration of symptoms was 24.40 ± 12.54 months; the majority of patients had experienced symptoms for 13-24 months (34.4%) or 25-36 months (29.2%). Asthma was the most common comorbidity, present in 48 patients (50.0%) when isolated and combined categories were considered together. CRSwNP was identified in 59 patients (61.5%) and CRSsNP in 37 patients (38.5%). Full baseline characteristics are presented in Table 1. 

**Table 1:** Baseline demographic and clinical characteristics (n = 96) CRSwNP: chronic rhinosinusitis with nasal polyps. CRSsNP: chronic rhinosinusitis without nasal polyps. SD: standard deviation.

Variable	Value
Age (years), Mean ± SD	39.96 ± 10.39
Male, n (%)	63 (65.6)
Female, n (%)	33 (34.4)
Duration of symptoms (months), Mean ± SD	24.40 ± 12.54
CRSwNP, n (%)	59 (61.5)
CRSsNP, n (%)	37 (38.5)

Presenting symptoms and anterior rhinoscopy findings

Nasal blockage was the most frequently reported symptom, present in 93 patients (96.9%), followed by nasal discharge in 85 (88.5%) and olfactory disturbance in 67 (69.8%). Recurrent cold, headache, and excessive sneezing were also common. On anterior rhinoscopy, nasal polyp or polypoidal mucosa was the dominant finding in 40 patients (41.7%), followed by oedematous or allergic mucosa in 28 (29.2%), polyp with mucopurulent discharge or edema in 19 (19.8%), and thick or mucopurulent nasal discharge in 9 (9.4%). Full symptom and rhinoscopy data are provided in Table 2.

**Table 2:** Presenting symptoms and dominant anterior rhinoscopy findings (n = 96) Symptoms represent multiple responses per patient (percentages do not sum to 100). Rhinoscopy findings indicate the single dominant finding recorded per patient.

Variable	n (%)
Nasal blockage	93 (96.9)
Nasal discharge	85 (88.5)
Disturbance of smell (hyposmia/anosmia)	67 (69.8)
Recurrent cold	56 (58.3)
Headache	49 (51.0)
Excessive sneezing	47 (49.0)
Snoring	38 (39.6)
Epistaxis	14 (14.6)
Earache	9 (9.4)
Facial pain/pressure	8 (8.3)
Ear fullness	5 (5.2)
Post-nasal drip	3 (3.1)
Cough	1 (1.0)
Rhinoscopy: Nasal polyp/polypoidal mucosa	40 (41.7)
Rhinoscopy: Oedematous/allergic mucosa	28 (29.2)
Rhinoscopy: Polyp with mucopurulent discharge/oedema	19 (19.8)
Rhinoscopy: Thick/mucopurulent nasal discharge	9 (9.4)

Baseline quality of life and endoscopic burden by CRS phenotype

Preoperative disease burden was significantly greater among patients with CRSwNP compared with CRSsNP. The mean preoperative SNOT-22 score was 45.20 ± 12.87 in CRSwNP versus 39.22 ± 11.71 in CRSsNP (p = 0.021). The mean preoperative Lund-Kennedy score was also significantly higher in CRSwNP than CRSsNP (9.92 ± 2.44 vs. 7.41 ± 2.49; p < 0.001), reflecting a greater objective endoscopic disease load in polypoidal CRS. These comparisons are shown in Table 3.

**Table 3:** Baseline scores by CRS phenotype Values are mean ± SD. Comparison between phenotypes used Welch's independent-samples t-test, which does not assume equal variances; degrees of freedom are therefore fractional. Exact p values were 0.0214 for SNOT-22 and 0.0000074 for Lund-Kennedy. CRSwNP: CRS with nasal polyps; CRSsNP: CRS without nasal polyps; SD: standard deviation; SNOT-22: 22-item Sino-Nasal Outcome Test.

Variable	CRSwNP (n = 59)	CRSsNP (n = 37)	t value (df)	p value
SNOT-22	45.20 ± 12.87	39.22 ± 11.71	t = 2.345 (df = 82.00)	0.021
Lund-Kennedy	9.92 ± 2.44	7.41 ± 2.49	t = 4.848 (df = 75.41)	<0.001

Change in SNOT-22 score after FESS

FESS was associated with a significant and statistically meaningful improvement in SNOT-22 scores at all postoperative time points (p < 0.001). The mean score dropped from 42.90 ± 12.72 preoperatively to 12.50 ± 7.98 at three months and 9.65 ± 6.33 at six months. The median score decreased from 44.0 before surgery to 11.0 at three months and 10.0 at six months. There was also a notable shift in severity categories: the proportion of patients with mild SNOT-22 burden (score ≤ 20) increased from 4.2% preoperatively to 83.3% at three months and 93.8% at six months. Conversely, those with severe burden (score > 50) decreased from 31.2% preoperatively to 0% at both postoperative assessments. These results are detailed in Table 4.

**Table 4:** Change in SNOT-22 score before and after FESS (n = 96) Overall mean score comparison used the Friedman repeated-measures test (χ² = 165.41, df = 2, exact p = 1.2 × 10^-36^). *Severity-category distribution compared using the chi-square test on the 3 × 3 contingency table (exact p = 3.8 × 10^-42^). FESS: functional endoscopic sinus surgery,  SNOT-22: 22-item Sino-Nasal Outcome Test.

SNOT-22 variable	Before FESS	3 months post-FESS	6 months post-FESS	Test statistic	p value
Mean ± SD	42.90 ± 12.72	12.50 ± 7.98	9.65 ± 6.33	χ² = 165.41 (df = 2)	<0.001
Mild (≤20), n (%)	4 (4.2)	80 (83.3)	90 (93.8)	χ² = 199.99 (df = 4)	<0.001*
Moderate (21–50), n (%)	62 (64.6)	16 (16.7)	6 (6.2)		
Severe (>50), n (%)	30 (31.2)	0 (0.0)	0 (0.0)		

Change in Lund-Kennedy endoscopic score after FESS

Objective endoscopic status showed significant and progressive improvement after FESS (p < 0.001). The mean Lund-Kennedy score decreased from 8.95 ± 2.74 at baseline to 3.61 ± 2.23 at three months (a 59.6% reduction) and further to 1.16 ± 1.42 at six months (an 87.1% reduction). The median score dropped from 9.0 before surgery to 3.0 at three months and 1.0 at six months. The greatest improvement occurred between baseline and three months, but continued progress between three and six months showed that endoscopic healing extended beyond the early postoperative period. Longitudinal Lund-Kennedy score data are shown in Table 5.

**Table 5:** Change in Lund-Kennedy endoscopic score before and after FESS p-value calculated using the Friedman repeated-measures test (χ² = 184.91, df = 2, exact p = 7.0 × 10⁻⁴¹). FESS: functional endoscopic sinus surgery

Time point	Mean ± SD	Change from baseline	Test statistic	p value
Before surgery	8.95 ± 2.74	Reference	χ² = 184.91 (df = 2)	<0.001
3 months after surgery	3.61 ± 2.23	59.6% reduction		
6 months after surgery	1.16 ± 1.42	87.1% reduction		

The simultaneous improvement in both SNOT-22 and Lund-Kennedy scores indicates a strong correlation between subjective symptom relief and objective mucosal recovery following FESS.

## Discussion

The present study included 96 patients with medically refractory CRS who underwent FESS. Postoperative evaluation demonstrated significant improvement in both patient-reported quality of life and objective endoscopic findings. The mean SNOT-22 score declined from 42.90 ± 12.72 preoperatively to 12.50 ± 7.98 at three months and 9.65 ± 6.33 at six months. Similarly, the mean Lund-Kennedy score improved from 8.95 ± 2.74 to 3.61 ± 2.23 at three months and 1.16 ± 1.42 at six months. These findings indicate that FESS resulted in sustained symptomatic improvement accompanied by progressive objective mucosal healing.

The demographic characteristics of this cohort were comparable with several previously published surgical CRS studies. The mean patient age was 39.96 years, indicating that the disease requiring surgical intervention predominantly affects individuals in early to middle adulthood. This observation was consistent with earlier reports by Sellami et al. (42.06 ± 12.47 years), Aghdas et al. (40.88 ± 16.11 years), and Nikakhlagh et al. (around 39 years) [19-21]. The male predominance of 65.6% in this cohort also matches the proportions of 64.4% and 62.2% reported by Aghdas et al. and Nikakhlagh et al., respectively [20,21].

The frequently reported presenting symptoms were nasal blockage, nasal discharge, and olfactory disturbance, which are characteristic manifestations of CRS. Nasal obstruction and discharge result from persistent mucosal inflammation and impaired sinus drainage, whereas loss of smell is particularly common in patients with nasal polyposis because of obstruction of the olfactory cleft and chronic inflammation. Similar findings have been described in previous studies, with Manohar et al. reporting nasal obstruction relief in 92.8% of patients after FESS, while Verma et al. documented improvement in nasal discharge in 86% of operated patients [22,23]. These findings support the clinical significance of the symptom profile observed in the present cohort and indicate why patients with longstanding obstruction and discharge were selected as potential surgical candidates.

The average symptom duration of approximately two years before surgery reflects the chronic nature of the disease in this cohort. Patients often adapt to long-standing nasal obstruction, facial pressure, and impaired olfaction, with progressive deterioration in quality of life resulting from ongoing inflammation and impaired mucociliary clearance. Begh et al. recommended that patients should demonstrate failure of at least three months of adequate medical therapy before undergoing FESS [24], while Sellami et al. adopted a comparable selection criterion [19]. In our study, the prolonged symptom duration likely reflects delayed presentation, delayed specialist referral, and extended unsuccessful empirical medical therapy, which are common in Indian tertiary care practice.

Asthma emerged as the most common comorbidity, while CRSwNP represented the predominant disease phenotype. This observation is consistent with the united-airway concept and the known link between nasal polyposis and lower airway inflammatory disease. Similar observations have been reported by Sellami et al., who described comorbidities such as asthma, respiratory allergy, and aspirin intolerance in 63.1% of CRSwNP patients [19]. Likewise, Kurien et al. identified asthma and allergic rhinitis as more frequent among CRSwNP patients in an Indian prospective cohort [25]. The observation that 50% of patients with CRSwNP in the present study had asthma is therefore biologically plausible and consistent with previously published evidence.

Preoperative SNOT-22 and Lund-Kennedy scores were significantly worse in CRSwNP than in CRSsNP. This difference is clinically meaningful, as polypoidal disease characteristically produces more severe obstruction, olfactory impairment, diffuse mucosal edema, and a higher endoscopic disease load. Deal and Kountakis similarly reported worse baseline SNOT-20 scores in patients with polyps than in those without [26], and Begh et al. noted a greater magnitude of SNOT improvement postoperatively in the polyp group [24]. However, Li et al. reported significant SNOT-20 improvements in both CRSwNP and CRSsNP following FESS, with phenotype-specific differences in the pattern of improvement over time [27]. Collectively, these findings suggest that although polypoidal CRS carries a higher baseline burden, appropriately selected patients in both phenotype groups can achieve meaningful benefit from surgery.

The magnitude of SNOT-22 reduction observed after FESS was large and clinically significant. The mean 6-month postoperative SNOT-22 score of 9.65 ± 6.33 in the present study is closely comparable to the 6-month value of 9.35 ± 8.93 reported by Sellami et al. in CRSwNP patients [19]. Begh et al. reported a decline from 54 ± 8.05 preoperatively to 12.9 ± 8.05 at 6 months [24], and Poojitha et al. documented improvement from 41.28 ± 15.30 to 6.83 ± 5.96 following FESS [28]. Aghdas et al. also demonstrated significant SNOT-22 improvement in CRSwNP patients, though their 12-month postoperative score was higher than that in the present study [20]. Overall, these comparisons support the conclusion that FESS yields clinically meaningful improvement in disease-specific quality of life, with the magnitude influenced by baseline severity, phenotype, postoperative care, and follow-up duration.

The distribution of symptom severity further supports the effectiveness of FESS in improving patient-reported outcomes. Based on changes in SNOT-22 scores, most patients who initially had severe symptoms experienced marked improvement after surgery. Preoperatively, 95.8% of patients were classified as having a moderate or severe symptom burden, and by 6 months, 93.8% had been reclassified to the mild category, with no patient remaining in the severe category. Comparable findings have been reported by Sharma et al., who observed an 83.67% improvement in SNOT-22 after FESS with a postoperative mean score of 6.83 at 3 months [29], and Poojitha et al., who demonstrated a similar shift toward low postoperative symptom burden [28]. These observations indicate that patients with greater baseline symptom severity often achieve the largest clinically meaningful improvements following surgical intervention, making the observed postoperative changes both statistically robust and clinically relevant [29].

A noteworthy point of the present study is the inclusion of objective endoscopic assessment alongside patient-reported outcomes. The mean Lund-Kennedy score declined by 59.6% at 3 months and 87.1% at 6 months, confirming substantial improvement in mucosal healing and supporting the symptomatic benefits reported by patients. Similarly, Poojitha et al. reported significant Lund-Kennedy score reductions following FESS [28], and Li et al. found significantly lower endoscopic scores at 3, 6, and 12 months compared with baseline in FESS-treated patients [27]. Kurien et al. emphasized the complementary value of using both SNOT-22 and Lund-Kennedy scoring to monitor subjective and objective improvement concurrently [25]. In contrast, Nikakhlagh et al. reported significant improvement in SNOT and CT scores but no statistically significant improvement in endoscopic scores [21]. Such variability across different studies reflects differences in sample size, baseline disease severity, postoperative regimen, and phenotype distribution.

The concurrent improvement in SNOT-22 and Lund-Kennedy scores indicates close agreement between patient-reported symptom relief and mucosal recovery in this study. This alignment is clinically important because subjective symptoms and endoscopic findings do not always improve simultaneously. Some patients continue to experience residual nasal obstruction, sleep disturbance, or olfactory dysfunction despite satisfactory endoscopic healing. However, in the present cohort, both subjective and objective outcomes improved together, supporting the routine use of these complementary assessment tools during postoperative follow-up.

The continued improvement in both SNOT-22 (from 12.50 to 9.65) and Lund-Kennedy (from 3.61 to 1.16) scores between 3 and 6 months is noteworthy. These findings indicate that recovery following FESS continues beyond the early postoperative period. Previous studies have similarly reported rapid initial improvement after restoration of sinus drainage, followed by gradual stabilization as postoperative edema resolves, mucosal healing progresses, and normal sinus ventilation is re-established. The sustained reduction in scores observed in our study reinforces the value of continued follow-up beyond the first postoperative assessment to evaluate long-term surgical outcomes accurately.

The comorbidity profile has practical implications for postoperative management. Patients with asthma and allergic airway disease require ongoing anti-inflammatory therapy and multidisciplinary coordination. Similarly, diabetes mellitus may influence prescribing decisions regarding corticosteroids, modify infection risk, and affect wound healing. Therefore, the high prevalence of associated comorbidities in our study highlights the importance of interpreting FESS outcomes within the broader context of each patient’s overall systemic health rather than considering sinonasal disease in isolation.

The present study also underscores the value of serial postoperative endoscopic assessment. The continued decline in Lund-Kennedy scores between three and six months indicates ongoing mucosal recovery after surgery. Scheduled endoscopic follow-up facilitates postoperative debridement, optimization of topical medical therapy, and timely detection of complications such as synechiae, recurrent nasal polyps, or persistent mucosal edema. Accordingly, postoperative endoscopic surveillance should be regarded as an essential component of comprehensive postoperative care rather than simply a method of documenting surgical results.

The findings are particularly relevant for tertiary care centers that frequently encounter patients with prolonged symptom duration. Repeated medical therapy without objective reassessment may delay definitive treatment in such patients. The results of the present study suggest that FESS can provide meaningful clinical improvement even in patients with a long duration of symptoms, provided that persistent chronic rhinosinusitis is confirmed by objective clinical and endoscopic evidence. However, this observation should not be interpreted as support for delaying surgery unnecessarily, as prolonged disease may continue to impair quality of life and contribute to ongoing mucosal inflammation before appropriate intervention is undertaken.

Three important clinical implications arise from the findings of this study. First, patients with chronic rhinosinusitis with nasal polyps (CRSwNP) generally present with a greater preoperative symptom burden and more severe endoscopic disease than those with CRSsNP. This information should be discussed during preoperative counseling to establish realistic expectations regarding treatment outcomes. Second, carefully selected patients can anticipate substantial and sustained improvement after FESS, although recovery continues progressively over several months. Third, postoperative evaluation should integrate both subjective and objective outcome measures rather than relying on a single assessment tool. While the SNOT-22 questionnaire reflects the patient’s perception of symptom severity and quality of life, the Lund-Kennedy endoscopic score objectively evaluates postoperative mucosal status and helps identify discrepancies between clinical symptoms and endoscopic healing.

There are several limitations of the present study that should be acknowledged. This was a nonrandomized, single-center study conducted without a parallel control arm of medically managed patients, limiting the ability to distinguish the effects of surgery from the natural course of disease, placebo effects, or continued postoperative medical therapy. Follow-up was limited to six months, preventing long-term reassessment of disease control and recurrence. Surgical management was individualized according to each patient’s clinical presentation, reflecting routine clinical practice with variability between participants. In addition, objective olfactory testing was not performed, despite olfactory dysfunction being a major symptom of chronic rhinosinusitis. Nevertheless, the study employed validated outcome measures, evaluated both CRSwNP and CRSsNP separately, and incorporated serial postoperative assessments of SNOT-22 and Lund-Kennedy scores. The simultaneous evaluation of patient-reported outcomes and objective endoscopic findings provides clinically meaningful evidence that is directly applicable to the management of CRS in tertiary care settings.

Future studies should incorporate measures such as CT scoring, analyze SNOT-22 subdomains, compare primary and revision cases, identify clinical predictors of persistent or recurrent symptoms following surgery, and extend follow-up to 12 months or beyond. Objective olfactometry and measures of asthma control would add further value in polyp-predominant cohorts. These additions would help identify patients who are most likely to benefit from surgery and those who may require more aggressive postoperative anti-inflammatory management.

## Conclusions

Functional endoscopic sinus surgery marked lasting gains in disease-specific quality of life and objective endoscopic status in carefully selected patients with chronic rhinosinusitis who had not responded to medical treatment. Nasal obstruction, discharge, and reduced sense of smell were among the most common complaints at presentation, and CRSwNP outnumbered CRSsNP in this group. Baseline scores showed that patients with CRSwNP started with higher SNOT-22 and Lund-Kennedy values, pointing toward heavier symptoms and more severe disease on endoscopy. Postoperative recovery was substantial. Mean SNOT-22 scores dropped significantly by three months and continued to decline through six months; most patients who began in the moderate or severe range were ultimately classified as mild. Lund-Kennedy scores followed the same trajectory, confirming that the mucosa had mostly healed. For CRS patients who were still symptomatic despite proper medical therapy, FESS is a sound surgical choice. Tracking SNOT-22 alongside the Lund-Kennedy score across follow-up visits allows clinicians to judge patient recovery.
